# Antimicrobial use–related problems and their costs in surgery ward of Jimma University Medical Center: Prospective observational study

**DOI:** 10.1371/journal.pone.0216770

**Published:** 2019-05-17

**Authors:** Gosaye Mekonen Tefera, Beshadu Bedada Feyisa, Tsegaye Melaku Kebede

**Affiliations:** 1 Department of Pharmacy, Clinical pharmacy course unit, Ambo University, Ambo, Ethiopia; 2 Department of public health, Nutrition course unit, Ambo University, Ambo, Ethiopia; 3 School of Pharmacy, Department of clinical pharmacy, Jimma University, Jimma, Ethiopia; Aga Khan University - Kenya, KENYA

## Abstract

**Introduction:**

Antibiotics are among the most commonly misused of all drugs, which results in antibiotic resistance and waste of resources and it has not been studied in Ethiopia. Therefore, this study was carried out to assess antibiotic use–related problems and their costs among patients hospitalized at the surgical ward of Jimma University Medical Center.

**Methods:**

Hospital-based prospective observational study was used to assess the prevalence, cost, and determinants of antibiotic use–related problems; multiple stepwise backward logistic regression analysis was done for a P value of < 0.25 to look for predictors of antibiotic use-related problems. Written informed consent was obtained and confidentiality was secured.

**Results:**

Among 300 participants, antibiotic use–related problems (ABURPs) were found in 69.3% of the study participants. The direct total cost attributed to these problems was approximated to a minimum of 2230.15 US$. Independent predictors for antibiotic use–related problems were: indication for antibiotic use like: use of antibiotic for prophylaxis; p < 0.0001, antibiotic use for both therapeutic & prophylaxis; p < 0.0001, CDC wound class I and II; p = 0.016 and; p = 0.002 respectively, overall poly-pharmacy and greater than 2 antibiotic exposure during hospital stay; p = 0.019and p = 0.006 respectively and hospital stay for ≥21 days; p = 0.007.

**Conclusion:**

The prevalence of antibiotic use-related problems was high and resulted in extra cost. Antibiotic use for prophylaxis, prophylaxis, and treatment, poly-pharmacy, greater than 2 antibiotic exposures during the hospital stay, CDC wound class I and II, and duration of hospital stay of ≥ 21 days was found to be independent predictors of antibiotic use–related problems.

## Introduction

Surgery is an essential component of health care [1]. Antimicrobials are used as prophylaxis and/ or therapeutic agents in surgery ward[2, 3], but indiscriminate uses is the main contributor to the emergence of resistant microbial strains[4, 5]. Currently (1987 to 2017), drug development programs seem insufficientto provide therapeutic cover for this resistantorganism[6–10]. In addition to World Health Organization (WHO), Center for Disease Prevention and Control of America (CDC) strongly recommends that governments should focus on control and prevention efforts through promoting rational antibiotic use, particularly in health care facilities to limit the spread of multi-drug resistant strains and reduce the generation of antibiotic-resistant bacteria [11–14].

Nevertheless, the use of these drugs in clinical practice has changed the natural course and improved the prognosis of infectious diseases. This is if and only if, antibiotics are used appropriately by the medical community[2, 3, 15–17]. Inappropriate antibiotic use may result in failure of therapy, morbidity, mortality and antimicrobial resistance as well as extra cost[18, 19]. From those commonly used medications, antimicrobial agents share higher percentages of misused drugs. Different studies witnessed that this problem was prevalent both in developed and developing countries, including Ethiopia, even though there was a difference in magnitude[20–30]. There are a lot of contributing factors for antibiotic use-related problems. To mention some; lack ofclinicians/surgeons motivation to practice as guidelinerecommendations, the absence of well-defined protocols, poor knowledge of prophylactic protocols, andthe use of previous personal experience[17, 31]. If this problem is not tackled early, common infections and minor injuries can be a *“catastrophic*” or “*big risk of terrorism*”; to human beings, is a very real possibility for the 21^st^ century[32].

Therefore, collecting data on inappropriate antimicrobials use is the first step in managing inappropriate antibiotic use. There are no robust studies that have addressed the antibiotic use-related problem and contributing factors, especially at the surgical wards. The investigators, therefore, conducted this study to assess antibiotic use-related problems and its economic consequence in JUMC, with the following objectives: (1) To assess the prevalence of antibiotic use-related problems at the surgical ward, (2) To determine predictors for antibiotic use-related problems at the surgical ward, (3) To evaluate direct out of pocket cost/extra cost of medication incurred due to irrational use of antibiotic at the surgical ward from the patients and the government perspective.

## Methods and materials

### Study area and period

The study was conducted from April 24 to July 24/2017 at Jimma University Medical Center (JUMC). It is, located 345 km southwest of Addis Ababa, the capital of Ethiopia. Surgery department has been run by 8 seniors, 43 residents, 5 general practitioners, and medical interns as rotation and provides services approximately for 5060 patients annually.

### Study design

A hospital-based prospective observational study was used.

### Source population

All patients who were admitted to the surgical ward for surgery case (elective or emergency).

### Study population

Patients who were admitted to the surgical ward for surgery and who were on antimicrobial or candidate for antimicrobial for treatment and/ or prophylaxis purpose during the study period with inclusion criteria.

### Inclusion and exclusion criteria

Inclusion criteria:Patientswith age of ≥ 18 years admitted at surgical ward and on antimicrobial or candidate for antimicrobial at a time of data collection.

Exclusion criteria: Patients prescribed only topical antibiotic were excluded.

### Sample size and sampling technique

Sample size (n) was calculated by using a single population proportion formula, which provided 247 as a minimum sample size for estimation of true proportion as follow:

$$n={Z\alpha /2}^{2}*\frac{P(1-P)}{W2}=\frac{1.96^{2}*0.75*0.25}{0.05^{2}}=288$$

Where;

1. P is the proportion of antibiotic use-related problem which is *0*.*75* from reference[28], Z is level of confidence = 1.96 with 95% CI
2. N is the size of the population that the sample is to represent = 1265 per three month
3. W is the amount of error that the researcher is willing to accept (margin of error) = 5%
4. Since N is less than 10,000 correction formula $nf=\frac{n}{1+\frac{n}{N}}=235$
5. 5% for non-response rate = 12 +235 = 247

Surveillance by using a consecutive type of sampling technique was used to collect data from 300 patients to make the data more robust.

### Study variables

#### Dependent variable

Antibiotic use-related problem (ABURPs).

#### Independent variables

Patient-related factors:Age, sex, socioeconomic status, level of education, marital status, smoking status, and patients medication-taking behaviors.

Disease-related factors:Types of surgery (elective and emergency), Type of wound class, type of procedure, ASA class, Comorbid conditions, Duration of hospital stay before and after surgery, Duration of operation and Charlson comorbidity index (CCI).

Drug-related factors:poly-pharmacy and Dosage Regimen.

Healthprofessional /facility-related factors: Timing of SAP administration, Duration of treatment and prophylaxisandAvailability of preferred antibiotic for a specific condition.

### Data collection instrument

Semi-structured questionnaires (English version) were used; to extract information from the patients and medical records (S 1 file). Some parts (the one that was used directly to collect information from patients or attendants like socio-demographic, compliance, informed consent, and patient information sheet) of the questionnaires were translated to AfanOromo and Amharic and back to English by a different person.

### Data collection process and management

All patients included in the study were followed daily from the time of admission for surgery until discharge. The distinction between prophylactic and therapeutic use of antimicrobial was differentiated after discussion with attending surgeon for post operation antibiotic use. Then the data collectors reviewed and filled patient’s information from the patient chart and through an interview.

#### Outcome measure

According to Robert J. Cipolle textbook of pharmaceutical care practice (third edition), there were 4 patient’s drug-related needs; if those needs were unfulfilled, they end up with seven basic categories of drug therapy problems [33]. The antibiotic use-related problem was identified by evaluating antibiotic use against different most recent international guidelines; such as American Society of Health-System Pharmacists (ASHP 2013), World Health Organization (WHO 2016), Infectious disease Society of America (IDSA) antimicrobial prophylaxis and therapeutic guidelines, and Ethiopian standard treatment guideline 2014. The ‘Medscape online drug interaction checker’, ‘Hippocrates online drug interaction checker’ and ‘Micromedex’ were used in order to detect whether drug interactions between the concurrently used medications exist or not, and classified under antibiotic use-related problem only if it was serious and contraindicated. Economic outcome or extra cost due to ABURPs was calculated for the direct cost of unnecessarily used antibiotic, i.e. when it was not indicated, high dose, prolonged duration, laboratory & treatment cost for antibiotic-related adverse reaction, overlapping and use of intravenous (IV) antibiotic while oral (PO) was appropriate[34]. The cost for this antibiotics was taken from JUMC, pharmacy cost list and for change in cost during the study period was solved by taking the average cost for that specific antibiotic.

### Data quality control

Training was given for 4 data collectors for 2 days before data collection. A panel of experts (clinical pharmacists and one senior surgery resident) assessed whether the data collection form will measure what it was intended to measure and if it was comprehensive enough to collect all the information needed to address the purpose and goals of the study. Then a pilot test for 15 patients (5% of sample size) was done and appropriate changes were made based on expert opinion. Before entry to EPIDATA manager version 4.0.2.101and analysis using statistical software package (SPSS) version 20.0, data was coded, cleared, categorized, compiled, checked for completeness and accuracy of data.

### Data processing and statistical analysis

All statistical tests were performed using SPSS. Descriptive statistics were used to summarize patients’ baseline characteristics, the prevalence, and types of ABURPs. Bivariateanalysiswasconducted to see the association between antibiotic use related problems and factors associated with it. A multivariable backward logistic-regression model was created for bivariate analysis results of P-value <0.25, to determine independent predictors for antibiotic use related problems throughmultiple stepwise backward logistic regression analysis. Probability values less than 0.05 was accepted as statistically significant.

### Ethical approval and participant’s consent

The ethical clearance was obtained from Jimma University, Institute of Health, Institutional Review Board approved the study under protocol number IHRPGQ/103/207. In addition, permission was sought from the respective heads of the Department of Surgery to conduct the study at the surgery ward. After relevant information was given on the research purpose and process, written informed consent was obtained from participants, and confidentiality was secured. The antibiotic use related problems identified during the data collection were handled by the investigators for resolution, to protect the patient from any potential risks or harms.

### Operational and standard definitions

Surgical Antimicrobial Prophylaxis: antimicrobial use for the issue of infection prevention in the absence of confirmed or suspected infection at the time of initiation[3]. Therapeutic antimicrobial use: antimicrobial use for the purpose of treating suspected or confirmed infection[3]. Poly-pharmacy:patientswho were prescribed or used more than or equal to five drugs concurrently [28]. Co-morbid condition:is a diseases condition when a patient has at least two diseases. Antibiotic use-related problem: is equivalent to drug therapy problem definition[33]with slight modification for this study.

## Results

### Sociodemographiccharacteristics of study participants

A total of 300 patients were included in the study, with 100% response rate. The mean (± SD) age of the participant was 42.62 ± 18.29 and the majority of patients (38%) were in the age range of 18 to 34 years. Majority of the study participants were male (67%), married (2/3), attended formal education (67.3%), live in rural area (55.3%), farmer (37.7%), earn less or equal to 6000 Ethiopian birrs (3/4) and nonsmokers (88.3%) (Table 1).

**Table 1:** Socio-demographic characteristics of the study participants at JUMC, April 24 to July 24/2017, Ethiopia (N = 300).

Variables	Categories	Frequency	Percent
**Age in year**	18–34	114	38
	35–54	99	33
	> = 55	87	29
**Sex**	Male	201	67.0
**Marital status**	Single	73	24.3
	Married	196	65.3
	Divorced	12	4.0
	Widowed	19	6.3
**Educational level**	can’t write & read	98	32.7
	primary (1–8)	118	39.3
	secondary (9–12)	50	16.7
	tertiary (diploma& above)	34	11.3
**Place of residence**	Rural	166	55.3
**Occupation**	Unemployed	14	4.7
	Merchant	42	14.0
	Housewife	23	7.7
	Farmer	113	37.7
	Student	33	11.0
	daily labor	8	2.7
	gov’t employee	32	10.7
	Retired	18	6.0
	Driver	6	2.0
	Other*	11	3.7
**Monthly income**	no regular income	52	17.3
	< 1500	111	37.0
	1500–6000	111	37.0
	> 6000	26	8.7
**Current Smoker**	No	265	88.3

N.B: other*: carpenter, evangelist, tailor, private employee

### Clinical characteristics of study participants

As per American Society of Anesthesiologist (ASA), patient’s physical status was class I, in (39.7%) of patients and (73%) of study participants fall in low-risk class (0–2 score), per age-adjusted Charlson comorbidity index (CCI) scoring. Among 269 patients for whom surgery was done, (57.3%) of patients’ blood loss during surgery was recorded as ≤ 1500 ml. The median time of operation in minutes was 85. While the most common CDC wound class was class II and IV (32.3% each) (Table 2).

**Table 2:** Clinical characteristics of study participants at JUMC, April 24 to July 24/2017, Ethiopia.

Variables	Categories	Frequency	%
**ASA class(N = 300)**	I	119	39.7
	II	99	33.0
	III	49	16.3
	IV	33	11.0
**Charlson CI risk classification (N = 300)**	low risk (0–2 score)	219	73.0
	moderate risk (3–4 score	47	15.7
	high risk (> = 5 score)	34	11.3
**Comorbid condition (n = 300)**	Yes	131	43.7
	No	169	56.3
**Number of comorbid condition (N = 131)**	1	75	57.3
	2	41	31.3
	≥ 3	15	11.5
**Duration of operation (median)**	85 minute	-	-
**Type of admission**	Emergency	141	47.0
	Elective	159	53.0
**CDC wound class (N = 300)**	class I	56	18.7
	class II	97	32.3
	class III	36	12.0
	class IV	97	32.3
	not applicable	14	4.7
**Amount of blood loss during surgery(N = 269)**	< 1500 ml	155	57.62
	≥1500 ml	4	1.49
	Unknown (not recorded)	110	40.89

N.B: **CI**- co-morbidity index, **CDC**- Centers for Disease Control and Prevention, **ASA**- American Society of Anesthesiologists

### Medication information of study participants

The most common reason for antibiotic use was the therapeutic/presumptive purpose in 67.7% of cases among 300 patients on an antibiotic during the study period. Twenty percent of patients had a history of antibiotic exposure within the past three months before admission. Majority of patients were exposed to ≤ 2 antibiotics (77.4%) during the total hospital stays. The mean antibiotic exposure during the study period was 2.02 ± 1.031. The prevalence of Poly-pharmacy in the study participants accounts for 21.7% (Table 3).

**Table 3:** Patients’ medication information among patient admitted to the surgery ward of JUMC, 24 April to 24 July 2017, Ethiopia.

Variables	Categories	Frequency	%
**Indication for antibiotic use (N = 300)**	therapeutic/presumptive only	158	52.7
	Prophylaxis only	97	32.3
	both (prophylaxis and therapeutic)	45	15.0
**History of antibiotic exposure in the past 3 months (N = 300)**	Yes	60	20.0
	No	180	60.0
	Unknown	60	20.0
**Poly-pharmacy status (N = 300)**	Yes	65	21.7
	No	235	78.3
**Total antibiotic exposure in hospital (N = 300)**	≤ 2	232	77.4
	≥ 3	68	22.6
	Mean (SD)	2.02 (1.031)	

### Prevalence of ABURPs among study participants

**Fig 1:**
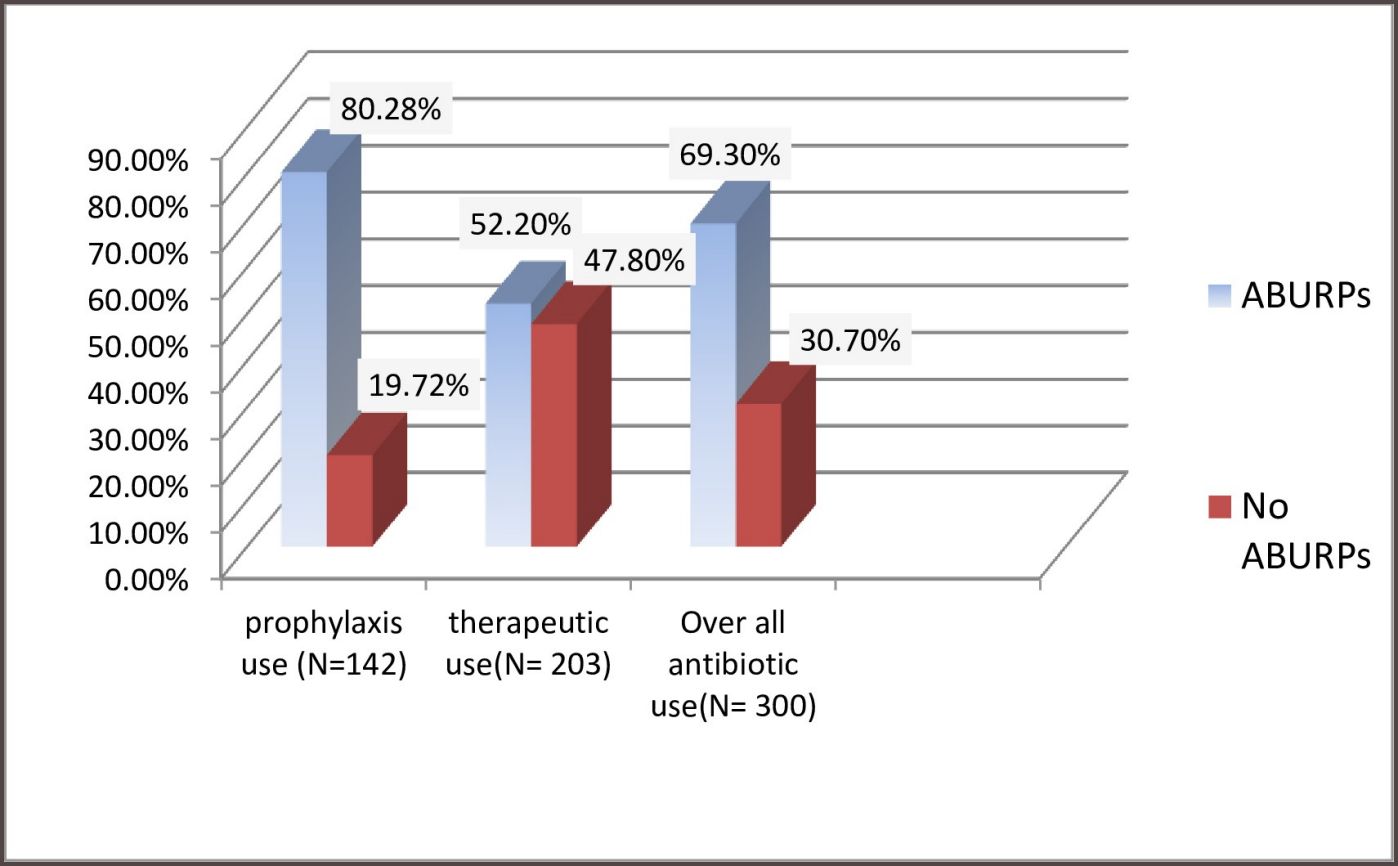
Prevalence of ABURPs among patients admitted at surgery ward of JUMC April 24 to July 24/2017 Ethiopia. N.B: ABURPs- antimicrobial use related problems.

ABURPs were found in 69.3% of the study participants, of which 80.28% wereSAP use-related problem and 52.2% was related to therapeutic antibiotic use. Among patients who were on SAP, only 28 (19.72%) used antibioticaccording to ASHP 2013 guideline recommendation (Fig 1). A total of 347 ABURPs were identified from 300 patients during the study period, with an average of 1.16 ABURP per patients (Table 4).

**Table 4:** Types and causes of antibiotic use-related problems identified at the surgical ward of JUMC from 24 April to 24 July 2017, Ethiopia.

Types of ABURPs	Cause of ABUR problems	No. of ABURPs in the prophylaxis	No. of Therapeutic ABURPs	Total	%
**Unnecessary antibiotic**	No medical condition	12	10	59	17.0
	Overlapping effect	1	29		
	Non-pharmacologic (no need for SAP) preferred	7	0		
**Need additional antibiotic**	Prophylaxis needed	2	0	17	4.9
	Additive or synergistic needed	8	7		
**Need different antibiotic**	More effective product available	3	11	16	4.6
	Route not appropriate	2	0		
**Dose too low**	The wrong dose ordered	79	3	114	32.9
	Inappropriate frequency (longer)	4	11		
	Inappropriate duration (short)	0	2		
	The timing of SAP (too late or early)	15	0		
**Potential or actual ADR**	Undesirable effect	0	2	8	2.3
	Unsafe drug	2	0		
	DI lead to ADR	1	2		
	Contra-indication	1	0		
**Dose too high**	The wrong dose ordered	0	16	72	20.7
	Inappropriate duration (longer)	46	10		
**Non-compliance**	The patient didn’t understand important information/ not informed	2	2	19	5.5
	Can’t afford medication	2	10		
	Health professional forget to give	1	2		
**Unclassified ABURPs**	Late to change IV to PO	0	41	42	12.1
	Need monitoring	0	1		
**Total**		188	159	347	100
**Average number of ABURPs per patient**				1.16	-

**Key**: ABURPs = antibiotic use related problems, DI = drug interaction, ADR = adverse drug reaction, IV = intravenous, PO = per oral, SAP = surgical antibiotic prophylaxis.

### Types and causes of ABURP by an indication of use

Dose too low was the top ranking ABURPs (32.9%), followed by dose too high (20.7%). The top cause of ABURPs was: wrong low dose for dose too low, inappropriate longer duration for dose too high, a drug with overlapping effect for unnecessary antibiotic therapy, too late to change IV to PO for unclassified ABURPs, can’t afford medication for non-compliance and additive or synergistic needed for need additional antibiotic therapy. Of 188 ABURPs identified among patients on SAP, the first type of ABURP is dose too low 98/188followed by dose too high 46/188. In addition, out of 159 ABURPs identified among patients on therapeutic antibiotic use; unclassified ABURP (too late to change IV to PO and need monitoring), 42/159 and unnecessary antibiotic therapy, 39/159 was ranked first and 2^nd^, respectively (Table 4).

### Antibiotics commonly involved in ABURPs

**Fig 2:**
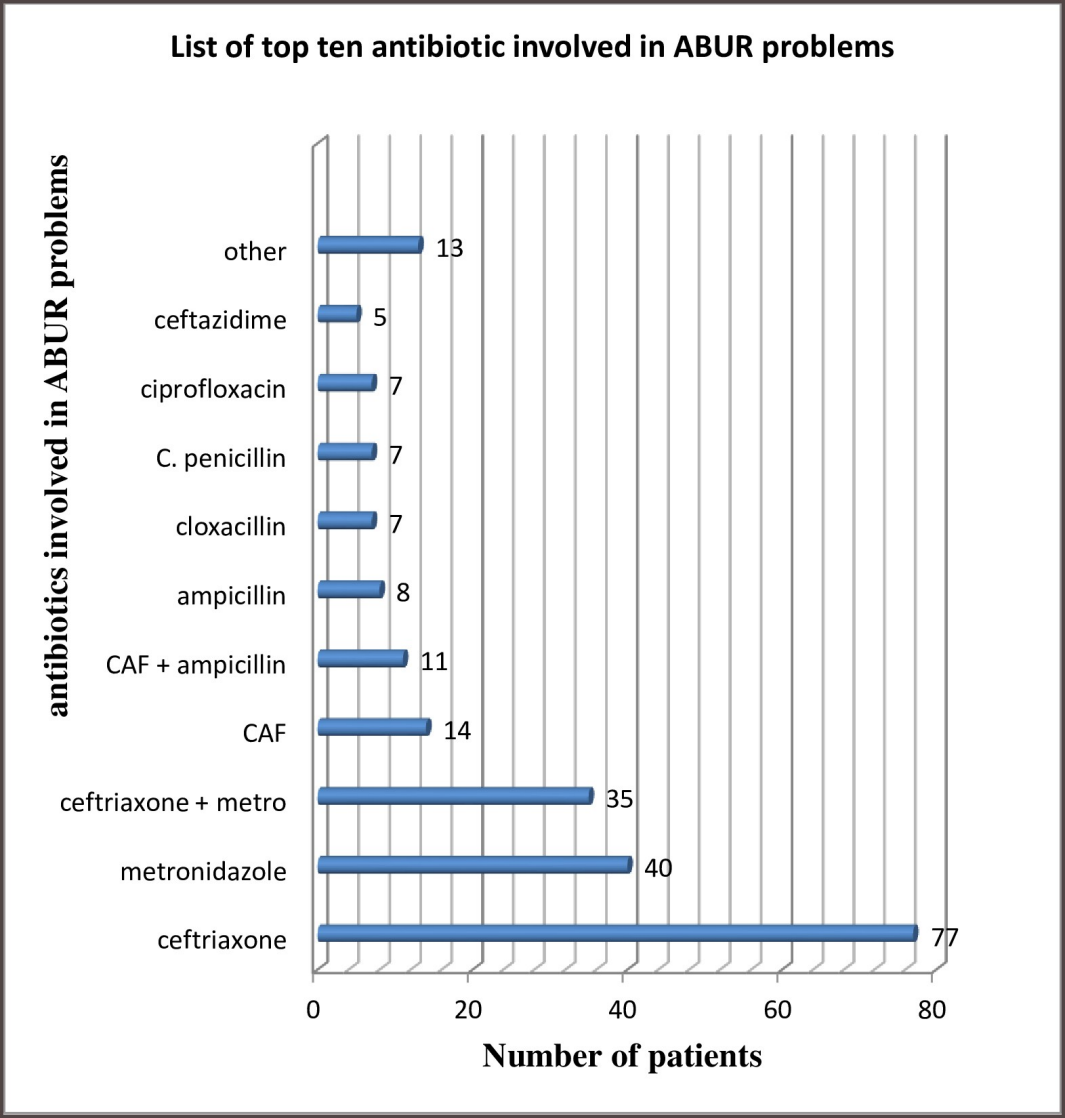
Top 10 antibiotics involved in ABURP during study period, JUMC, 2017 Ethiopia. N.B; other = amoxicillin, cephalexin, tinidazole, clindamycin, vancomycin, norfloxacin, CAF = chloramphenicol, c. penicillin = crystalline penicillin, + (Pluss) = concurrent use.

Ceftriaxone was the most commonly used antibiotic as well as the top ranking antibiotic involved in ABURPs in about 77 patients followed by metronidazole in 40 patients, among 208 patients with ABURPs (Fig 2).

### The economic consequence of ABURPs

Even though measuring the exact cost of ABURPs is difficult, it was tried to calculate the direct out of pocket cost for an antibiotic from both the patient and the governmental perspective; for self-payer and free, respectively. Accordingly, among 208 patients with ABURPs, 132 (63.46%) exposed to direct out of pocket extra cost due to ABURPs, of which 106 patients were self-payer while 26 were free. The mean (SD) extra cost of antibiotic was 17.21 US$(20.03) and (15.63 US$(7.81)) in self-payer and free groups, respectively, as calculated based on the cost of antibiotic of JUMC pharmacy cost list during the study period. The total cost attributed to direct ABURPs, among 132 patients in 3 month period was approximated to a minimum of 52,118.63 ETB (2230.15 US$), of which 1823.74 US$ was contributed by self-payer, while, 406.41 US$ was free (Table 5). A total of 8920.61 US$ per year, of which government can lose unnecessarily at least 1625.64US$ per year if we extrapolate the 3-month data assuming that no variation in disease condition and practice. From the patient perspective, it could be at least 7294.97 US$ per year, which is a great burden to society as well as to the government without adding any clinical benefit for the patient.

**Table 5:** ABURPs extra cost (direct cost for antibiotics) within 3 months (24 April to 24 July)2017 from the patient and government perspective, JUMC, Ethiopia.

cost of health care covered and direct cost incurred by ABURPs					
Perspective	Number of patients	Minimum cost/patient in ETB	Maximum cost/patient in ETB	Mean (SD) in ETB	Total cost in ETB
**ABURPs related extra cost for self-payer (N = 169)**	106	2.00	2936.1	402.08 (468.10)	42620.83
**ABURPs related extra cost for free (N = 39)**	26	37.90	671.78	365.30 (182.39)	9497.80
**Total**	132	2.00	2936.1	394.82(426.84)	52,118.63

**NB**. Cost is calculated based on average cost of antibiotic during the study period as claimed by JUMC pharmacy cost list, ABURPs–antibiotic use-related problems, ETB = Ethiopian birr, at a time of this report writing, September 02/2017, 1 US$ was equal with 23.37 ETB,

### Determinant factors for ABURPs

A stepwise backward multivariate logistic regression showed that, indication for antibiotic use, types or discipline of surgery, poly-pharmacy, total antibiotic exposure during hospital stay, CDC wound class, overall clinical outcome of patients, and the duration of hospital stay in days were found to beindependent predictors of antibiotic use related problems in multivariate logistic regression analysis.

Accordingly, indication for antibiotic use like: SAP use was about 7 times more likely to incur ABURPs [AOR, 6.834; 95% CI, 3.025–15.439; p < 0.0001) and both for therapeutic & SAP use was about 8 times more likely to incur ABURPs, [AOR, 8.211; 95 CI, 2.215–30.44; p < 0.0001] than antibiotic use for therapeutic purpose alone. Surgical discipline or disorder related to Cardiothoracic was about 96% time less likely to incur ABURPs [AOR, 0.040; 95 CI, 0.003–0.57; p = 0.018] than upper and lower GIT. Patients with CDC wound class I and II were about 15 times more likely to have ABURPs [AOR, 14.939; 95% CI, 1.646–135.560; p = 0.016] and [AOR, 33.555; 95% CI, 3.726–302.209; p = 0.002] respectively, than patients with no wound. Overall poly-pharmacy relative to patients with no poly-pharmacy and greater than 2 antibiotic exposure relative to less than 2 antibiotic exposure during hospital stay were about 3 times more likely to have ABURPs [AOR, 3.343; 95% CI, 1.224–9.133; p = 0.019] and about 5 times more likely to have ABURPs [AOR, 4.838; 95% CI, 1.586–14.752; p = 0.006] respectively. Referral was about 98% times less likely to incur ABURPs [AOR, 0.017; 95% CI, 0.001–0.327; p = 0.007] than patients whose clinical outcome was improved. Similarly, compared with those who stayed for less or equal to 20 days, patients that stayed for ≥ 21 days were about 4 times more likely to have ABURPs [AOR, 3.526; 95% CI, 1.416–8.782; p = 0.007] (Table 6).

**Table 6:** Multivariate logistic regression analysis for the determinants of antibiotic use-related problems among patients admitted to the surgical ward of JUMC, from 24 April to 24 July 2017, Ethiopia.

Variables	Categories	ABURPs		P value	AOR (95% CI)
		No (%)	Yes (%)		
**An indication of antibiotic use**	Therapeutic	67 (42.4)	91 (57.6)		1
	SAP	20 (20.6)	77 (79.4)	< 0.0001	6.834 (3.025 15.439)
	Both therapeutic + SAP	5 (11.1)	40 (88.9)	< 0.0001	8.211 (2.215–30.44)
**Types or discipline of surgery**	upper and lower GIT	31 (36.0)	55 (64.0)		1
	Breast	3 (50.0)	3 (50.0)	0.06	0.121 (0.013–1.089)
	Urology	12 (23.1)	40 (76.9)	0.464	1.738 (0.396–7.630)
	Cardiothoracic	7 (63.6)	4 (36.4)	.018	.040 (0.003–0.571)
	biliary tract	2 (28.6)	5 (71.4)	.558	.462 (0.035–6.106)
	head and neck	19 (46.3)	22 (53.7)	.180	.420 (0.118–1.491)
	neurosurgery	1 (11.1)	8 (88.9)	.182	.169 (0.012–2.299)
	skin and deep tissue	12 (18.5)	53 (81.5)	.603	1.317 (0.466–3.721)
	hernia repair	1 (16.7)	5 (83.3)	.522	2.229 (0.192–25.870)
	Other ^c^	4 (23.5)	13 (76.5)	.828	0.844 (0.182–3.912)
**CDC wound class**	Not applicable	5 (35.7)	9 (64.3)		1
	Class I	18 (32.1)	38 (67.9)	.016	14.939 (1.646–135.56)
	Class II	20 (20.6)	77 (79.4)	.002	33.555 (3.726–302.209)
	Class III	20 (55.6)	16 (44.4)	---	----
	Class IV	29 (29.9)	68 (70.1)	.198	2.578 (0.609–10.912)
**Poly-pharmacy**	No	81 (34.5)	154(65.5)		1
	Yes	11 (16.9)	54 (83.1)	.019	3.343 (1.224–9.133)
**antibiotic exposure**	≤ 2	85 (36.6)	147(63.4)		1
	≥ 3	7 (10.29)	61 (89.71)	.006	4.838 (1.586–14.752)
**Overall clinical out-come of patients**	Improved	73 (28.5)	183(71.5)		1
	LAMA	3 (27.3)	8 (72.7)	.202	.168 (0.11–2.608)
	Referral	8 (61.5)	5 (38.5)	0.007	.017 (0.001–0.327)
	Death in hospital	5 (29.4)	12 (70.6)	.911	1.110 (0.176–7.013)
	Not improved	3 (100.0)	0 (0.0)		
**Overall Hospital stay (days)**	≤ 20	66 (40.49)	97 (59.51)		1
	≥ 21	26 (19.0)	111 (81.0)	0.007	3.526 (1.416–8.782)

**N.B**: ABURPs–antibiotic use-related problems, LAMA- left against medical advice, GIT- gastrointestinal tract, SAP- surgical antibiotic prophylaxis, AOR- adjusted odds ratio, CI- confidence interval, other ^c^ = orthopedic, vascular surgery, joint (biopsy), joint and bone, joint surgery

## Discussion

It was found that overall ABURPs were 69.3% among study participants. Similar to this study, in a different area of the world antibiotic use related problems were reported with different prevalence; in Ethiopian University Hospitalsit ranges from 75.7% to 80.6%[28, 29];and73.6%in Malaysia[27]. This means in average at least 1.16 ABURP per each patient admitted to surgery ward of JUMC, which was lower than, a study in Singapore[26], a mean of 1.4 antibiotic prophylaxis errors per surgery. Among patients who were on SAP, only 28/142 patients used SAP according to ASHP 2013 guideline recommendation, which indicates surgical antibiotic use-related poor practice in JUMC. However, the problem was less when compared with the study done in Iran[25], only 1/ 155 surgical procedurewas correct.

The most frequent type of overall antibiotic use-related problem identified was dose too low, (32.9%) followed by dose too high (20%) in JUMC surgery ward, compared to study done in JUMC medical ward [28] needs additional drug therapy was a top ranking antibiotic use problem(29.6%) followed by ‘dose too low’ (28.9%); in Mekelle, Ethiopia[29]inappropriate duration of treatment (47.4%); in Malaysia[27] inappropriate timing (36.4%) was the top ranking antimicrobial use problem. This variation could be explained by patients in the medical ward might use antibiotic for treatment purpose unlike that of surgery (both treatment and prophylaxis). In addition, dose too low was more occurred in prophylaxis than in treatment, which was contributed by ceftriaxone; commonly used as 1 g for surgical prophylaxis. But, ASHP 2013 guideline[16] recommends 2 g of ceftriaxone for SAP.

Dose too high (20.7%) in this study was slightly higher than that of JUMC medical ward[28] (15.1%) because of unnecessary prolonged surgical prophylaxis was considered as dose to high which was common in surgery ward. However, it was lower than study in TikurAnbessa specialized hospital (TASH)[30]the use of high dose ceftriaxonewas(80.3%).

Hospitalized patients initially on intravenous antibiotics can be safely switched to an oral equivalent, within the third day of admission once clinical stability is established and for a disease that can be treated by the oral antibiotic[34]. Because, it has fewer complications, fewer healthcare costs, and earlier hospital discharge. However, a study done in Lebanese hospitals shows that IV antibiotic courses were only in one-third of the candidate were switched [35]. Similar to this in JUMC; too late to change IV to PO was 41/159 (25.8%), which was almost contributed by metronidazole. But, the cost of IV metronidazole was around 25 times costly than that of oral preparation without considering the cost for cannula and nursing time during the study period in JUMC. This has a great economic burden to the patient or government, especially in developing countries like Ethiopia where the budget is a constraint. This emphasizes an important gap in the field of conversion from intravenous to oral antibiotic therapy and the need for integration and reinforcement of the appropriate Antibiotic Stewardship Programs in JUMC. In Ethiopia, TASH, ceftriaxonewas not switched to oral in 66.2%[30]. This problem was usually due to a lack of information and previous personal experience [31].

In this study, the total cost attributed to direct ABURPs among 132 patients in the 3-month period was approximated to a mean (SD) 16.9 US$(18.26) and at least 2230.15 US$. The true extra cost related to ABURPs might be greater than what was mentioned above, because of the study was interventional and the cost of an instrument like IV set, laboratory and treatment costs for morbidity secondary to antibiotic use related problems were not included. Other studies reportwere almost similar to our study finding. But, still extra cost added to patients was higher than that of Iran[25]; this might be due to: the later study only considered prophylaxis cost and difference in cost of antibiotic that was involved in ABURP; as a result of change in time and total study population as well as study period deference. Study from Iran [25] non-adherence to SAP practice guidelines resulted in almost US$ 10 extra cost per patient or US$ 1527 extra cost for the 15-day period of the study, due to over-use of antibiotics. In another way[36], it was shown that reducing 24-hour prophylaxis to a single dose appropriate antibiotic, results in a potential monthly saving of US$ 2000. Workshop/ educational campaign and incorporation of a well-trained clinical pharmacist[37] and infectious disease specialist in the surgical ward of JUMC is paramount.

In this study, multivariate logistic regression analysis showed that indication for antibiotic use for SAP was 7 times, p < 0.0001 and dual indication (therapeutic & SAP) was 8 times,p< 0.0001 more likely to incur ABURPs, respectively. As other study indicated, the reason for this was related to the absence of well-defined guideline and poor knowledge of SAP protocols [31]. On the other hand, patients with CDC wound class I and II were significantly associate with the occurrence of ABURPs. This result was supported by antibiotic use for surgical prophylaxis was independent predictors of ABURPs. In addition, poly-pharmacy, >2 antibiotic exposure and a hospital stay of >20 days were significantly associated with ABURPs. Which was similarly reported in other studies like JUMC medical ward[38] and in Nigeria[39], because of an increase in drug interaction or unnecessary overlap, unaffordability and etc. Besides this, the longer the hospital stay, the more the total number of antibiotic exposure and the more hospital acquired infection which needs for treatment by antibiotic which could result in unaffordability.

## Conclusion and recommendation

The prevalence of ABURPs was high; especially in SAP use compared to therapeutic, which deserve rapid action to preserve those antibiotics in the market and to reduce the occurrence of surgical site infection (SSI). The most prevalent type of ABURPs was dose too low followed by dose to high. The consequences of irrational antibiotic use resulted in high extra cost. An indication of antibiotic use for prophylaxis, a dual indication (prophylaxis and treatment), poly-pharmacy, greater than 2 antibiotic exposures during a hospital stay, CDC wound class I and II, and duration of hospital stay for ≥ 21 days was found to be independent predictors for antibiotic use-related problems in the study area. To reduce the problem, government and stakeholders should prepare and implement the strategy that was found efficient and effective by research, in order to reduce inappropriate use of antibiotics and extra cost.

## Supporting information

S1 File Data abstraction tool. Attached as a separate file with title name of “supporting data/ data collection tool”.
(DOCX)

S1 Dataset 2 only antibiotic SPSS final gosi 300 for PlOS one 2019. (SAV)
