# Supplementary material for: Antimicrobial use–related problems and their costs in surgery ward of Jimma University Medical Center: Prospective observational study
Source: PLoS One. 2019 May 17;14(5):e0216770. doi: 10.1371/journal.pone.0216770 (PMC6524801; doi:10.1371/journal.pone.0216770)
Supplement: S1 File — Attached as a separate file with title name of “supporting data/ data collection tool”. (DOCX) [file pone.0216770.s001.docx]

**JIMMA UNIVERSITY, INSTITUTE OF HEALTH SCINCES, SCHOOL OF PHARMACY: DEPARTMENT OF CLINICAL PHARMACY**

Antibiotic use related problems, cost and its determinants among patients admitted to surgical ward of JUMC from April 24 to July 24/ 2017, Jimma Zone, Oromia region, South West Ethiopia.

**PART I: SOCIO-DEMOGRAPHIC CHARACTERISTICS**

| **Socio-demographic Characteristics** | **Values** |
| --- | --- |
| 1. Identification No. |  |
| 1. Age (years) |  |
| 1. Sex | a) Male b) Female  If female, I) pregnancy a) yes (with) trimester b) No  II) lactation a) Yes b) No |
| 1. Religion | a) Muslim b) Orthodox c) protestant d) catholic  e) Other (specify)………. |
| 1. Marital status | 1. Single…... b) Married………   c) Divorced…. D) Widowed……. |
| 1. Education level | 1. Can’t read and write……. 2. Primary (1-8)………. 3. Secondary (9-12)…… 4. Tertiary (diploma and above)…… |
| 1. Place of residence | a) Rural……… b) Urban…….. |
| 1. Occupation | \| a) Unemployed  c) House wife  e) Student  f) Daily labor…… \| b) Merchant  d) Farmer  g) gov’t employee  f) Other(s) [Specify]:____________ \| \| --- \| --- \| |
| 1. Income (monthly in ETB) | 1. No constant income c) 1500- 6000 2. < 1500 d)>6000 |
| 1. Smoking status (currently) | 1. Yes b) No |
| 1. ASA class | a) I b) II c) III d) IV |
| **N.B ASA** = American society of anesthesiologist physical status classification | |

**PART II: PATIENTS CLINICAL INFORMATION:**

1. Current Chief compliant……………………………………………………………………
2. Diagnosis (reason for being in surgical ward)………………………………………………………………………………………………………………………………………………………………………………………
3. Is there a comorbid condition? Yes…….. No……
4. If yes to question number 3, **Number of co morbidity**
5. </=3
6. >3
7. If yes to question number 3, list comorbid diseases [charlson comorbidity index; **CCI**)
8. ……………………..
9. ……………………..
10. ……………………..
11. …………………….
12. ……………………
13. indications for antibiotic therapy
14. therapeutic/ treatment
15. prophylactic
16. **site or type of surgery (Surgical disciplines**)
17. Upper & lower gastrointestinal
18. Breast
19. Urology
20. Cardiothoracic
21. Biliary tract
22. Head and neck
23. Neurosurgery (craniotomy)
24. Skin and deep tissue (incision, drainage, excision and graft )
25. Other (specify)…………………………………..
26. Types of admission
27. Emergency
28. Elective
29. Wound class (per CDC)
30. Class I
31. Class II
32. Class III
33. Class IV
34. Amount of blood loss during surgery (from operation note)
35. < 1500ml
36. >/= 1500ml
37. Unknown (note recorded) d) surgery note done
38. For how long the duration of surgery lasted (***in minutes***)?..................................
39. Any surgical antimicrobial prophylaxis intra operative re dosing? A) Yes…… B) No…….. B) not needed
40. If yes to question number 12, frequency or how many times re dosed?.........................................
41. Review of Systems and Laboratory values from day of admission to discharge

| \| **Signs, Symptoms, Lab values** \| \| --- \| | **List only deviation from normal and or relevant baseline values)** | | | | | | | | | | Remark |
| --- | --- | --- | --- | --- | --- | --- | --- | --- | --- | --- | --- | --- |
| **Date** |  |  |  |  |  |  |  |  |  |  |  |
| **VITAL SIGNS:** |  |  |  |  |  |  |  |  |  |  |  |
| Temp |  |  |  |  |  |  |  |  |  |  |  |
| BP |  |  |  |  |  |  |  |  |  |  |  |
| RR |  |  |  |  |  |  |  |  |  |  |  |
| HR |  |  |  |  |  |  |  |  |  |  |  |
| **CNS/NEUROLOGIC** |  |  |  |  |  |  |  |  |  |  |  |
| **EENT** |  |  |  |  |  |  |  |  |  |  |  |
| **CVD and Respiratory** |  |  |  |  |  |  |  |  |  |  |  |
| **FLUID & ELECTROLYTE** |  |  |  |  |  |  |  |  |  |  |  |
| Na+ |  |  |  |  |  |  |  |  |  |  |  |
| K+ |  |  |  |  |  |  |  |  |  |  |  |
| Ca++ |  |  |  |  |  |  |  |  |  |  |  |
| Cl- |  |  |  |  |  |  |  |  |  |  |  |
| **RFT** |  |  |  |  |  |  |  |  |  |  |  |
| Srcreatinine |  |  |  |  |  |  |  |  |  |  |  |
| BUN |  |  |  |  |  |  |  |  |  |  |  |
| CrCl |  |  |  |  |  |  |  |  |  |  |  |
| **LFT** |  |  |  |  |  |  |  |  |  |  |  |
| AST |  |  |  |  |  |  |  |  |  |  |  |
| ALT |  |  |  |  |  |  |  |  |  |  |  |
| ALP |  |  |  |  |  |  |  |  |  |  |  |
| Albumin |  |  |  |  |  |  |  |  |  |  |  |
| Bleeding |  |  |  |  |  |  |  |  |  |  |  |
| **GI** |  |  |  |  |  |  |  |  |  |  |  |
| **GU/REPRODUCTION** |  |  |  |  |  |  |  |  |  |  |  |
| Urine Analysis |  |  |  |  |  |  |  |  |  |  |  |
| **ENDOCRINE** |  |  |  |  |  |  |  |  |  |  |  |
| Glucose |  |  |  |  |  |  |  |  |  |  |  |
| T4, T3, TSH |  |  |  |  |  |  |  |  |  |  |  |
| **MSK** |  |  |  |  |  |  |  |  |  |  |  |
| **DERMATOLOGY** |  |  |  |  |  |  |  |  |  |  |  |
| **HEMATOLOGY** |  |  |  |  |  |  |  |  |  |  |  |
| WBC |  |  |  |  |  |  |  |  |  |  |  |
| RBC |  |  |  |  |  |  |  |  |  |  |  |
| Neutrophils |  |  |  |  |  |  |  |  |  |  |  |
| Hgb |  |  |  |  |  |  |  |  |  |  |  |
| MCV |  |  |  |  |  |  |  |  |  |  |  |
| Platelets |  |  |  |  |  |  |  |  |  |  |  |
| INR/PT |  |  |  |  |  |  |  |  |  |  |  |
| **Culture (if available)** |  |  |  |  |  |  |  |  |  |  |  |
| **Other important lab. Result** |  |  |  |  |  |  |  |  |  |  |  |

**PART III: PATIENTS MEDICATION INFORMATION**

1. Cost of health care covered: A, self-payment B, free
2. Extent/pattern of antimicrobial use according to indication for therapy (**N.B if there is change in either dose or route or frequency or any other consider as new prescription and rewrite on new space)**

| Serial No. | Medications | Indication (disease) | Dose | Frequency | Route | Timing if SAP (minutes) | Started on (D/M/Y) | D/C (D/M/Y) |
| --- | --- | --- | --- | --- | --- | --- | --- | --- |
| 1 |  |  |  |  |  |  |  |  |
| 2 |  |  |  |  |  |  |  |  |
| 3 |  |  |  |  |  |  |  |  |
| 4 |  |  |  |  |  |  |  |  |
| 5 |  |  |  |  |  |  |  |  |
| 6 |  |  |  |  |  |  |  |  |
| 7 |  |  |  |  |  |  |  |  |
| 8 |  |  |  |  |  |  |  |  |
| 9 |  |  |  |  |  |  |  |  |
| 10 |  |  |  |  |  |  |  |  |
| 11 |  |  |  |  |  |  |  |  |

**N.B:** SAP-surgical antimicrobial prophylaxis before skin incision

**D/C= discontinued**

1. Antibiotic use in past 3 months: A, Yes……. B, No…… C, unknown
2. History of antibiotic allergy: A, Yes……. B, No……. C, unknown
3. Any pre-admission medications (all including antimicrobial and other drugs)

a) Yes……. b) No…… c) Unknown

1. If yes to question No 5. Number of current medications

a) Antimicrobial……….. b) Other drugs)…………..

1. Overall number of drugs used during hospital stay, [**antibiotic exposure**]

a) Antimicrobials………………….. b) Other drugs……………….

**PART IV: ANTIBIOTIC USE RELATED PROBLEMS (ABURPS) IDENTIFIED**

1. **Non adherence** a) Yes b) No
2. If yes for above question, reason for non-adherence
3. The patient is not instructed or does not understand important information regarding his medications
4. Not willing to take medication
5. Can’t afford medication
6. Health professional’s forget to give the drug
7. Is there a drug-drug interaction? a) Yes b) No
8. If yes to above question (No 1), list those drugs involved in drug-drug interaction clearly …………………………………………………………………………………………………………………………………………………………………………………………………
9. If yes to above question (No 1), level of interaction (per Medscape and /or Micromedex and /or Hippocrates drug interaction checker),

a) Contra-indicated b) Serious c) Significant d) Minor

1. Assessment of Antimicrobial therapy appropriateness? **N.B 1 (**over all therapy, if single deviation from what is recommended for each individual antimicrobial eg. Frequency considered as irrational. **N.B** 2 check ABURPS in their sequence of **indication→ efficacy→ safety→ compliance**)
2. Rational
3. Irrational
4. If irrational (No 4), state the drug used irrationally clearly ……………………………………………………………………………………………………………………………………………………………………………………………………..
5. If irrational (for question 4) fill Type and Number of Specific ABURPS Category by encircling on one of the list)
6. Unnecessary drug therapy
7. No medical condition
8. Treating avoidable ADR
9. Drug which have overlapping effect
10. Needs additional drug therapy
11. Untreated indication
12. Preventive or prophylactic
13. Synergistic or potentiating action
14. Needs different drug product
15. More effective drug available
16. Route of administration is not the appropriate to treat condition (lead to sub therapeutic at site of infection)
17. The dosage form of the drug product is inappropriate
18. Dosage too low
19. Wrong dose
20. Frequency inappropriate (longer frequency)
21. Inappropriate duration ( too short duration)
22. Drug interaction lead to low dose
23. Timing of prophylaxis antibiotic is too early for specific antimicrobial
24. Timing of prophylaxis antibiotic is too late ( after skin incision)
25. Adverse drug reaction
26. Undesirable effect
27. Unsafe drug for patient
28. Drug interaction reduce dose
29. Contraindication present
30. Wrong route of administration which is un safe for patient ( eg Iv ceftriaxone which contain lidocaine)
31. The dosage regimen was changed too rapidly
32. Dosage too high
33. Wrong dose prescribed or administered
34. Wrong dose administered while prescription is wright
35. Frequency inappropriate (short frequency)
36. Duration inappropriate (longer duration of therapy)
37. Drug interaction which is non-dose dependent
38. Incorrect administration (bolus for infusion drug)
39. Drug dosage not adjusted to renal function in renal impairment
40. Other problems (unclassified antibiotic use related problems
41. Non adherence with formulary/ guideline (STG, IDSA, ASHP, WHO etc.)
42. Too late to change IV to PO medication (per patient/ drug condition eg Po absorption, clindamycin , quinolone, metronidazole)
43. Need for additional or more frequent monitoring (**list eg CBC, RFT, LFT etc)**
44. Need for additional diagnostic test like………..
45. Estimated direct out of pocket cost from patient perspective and government for antibiotic use related problem [for patient who self-payments and free respectively)

| Reason for additional cost | List the name of the item used (name of drug, lab or diagnostic) | Cost/unit (to the lowest unit)  Eg. Cost of amoxicillin 500mg/capsule | Number of unit used | Cost incurred |
| --- | --- | --- | --- | --- |
| **For treatment of morbidity** |  |  |  |  |
|  |  |  |  |  |
|  |  |  |  |  |
|  |  |  |  |  |
|  |  |  |  |  |
| Unnecessary **prolong antimicrobial** use or **late IV to PO** change |  |  |  |  |
|  |  |  |  |  |
|  |  |  |  |  |
|  |  |  |  |  |
|  |  |  |  |  |
| **For lab investigation** |  |  |  |  |
|  |  |  |  |  |
|  |  |  |  |  |
|  |  |  |  |  |

1. **Fill the following day in day/month/year:**

A, Admission day …………………………………………………...

B, surgery day (if done with procedure)……………………………………………...

C, discharged day (if discharged at end of study)………………. …..

1. **Over all Clinical outcomes of patient**
2. Surgical site infection for patient undergoing surgery
3. Cured/improved and discharged
4. Left against medical advice (LAMA)
5. Referral
6. Note improved and discharged
7. Death in hospital, (specify possible cause) ……………………………………………………………………………..

**PART V: ADVERSE DRUG REACTION MEASUREMENT TOOL (NARANJO SCALE)**

1. **Actual ADR a) Yes b) No**
2. If yes to above question, please answer the following question and give pertinent score for patient with suspected adverse drug reaction only

| **s.no** | **Questions** | **Yes** | **No** | **Don’t know** | **Score** |
| --- | --- | --- | --- | --- | --- |
| **1** | Are there previous conclusive reports on this reaction? | **+1** | **0** | **0** |  |
| **2** | Did the adverse event appear after the suspected drug was administered? | **+2** | **-1** | **0** |  |
| **3** | Did the adverse reaction improve when the drug was discontinued or a specific antagonist was administered? | **+1** | **0** | **0** |  |
| **4** | Did the adverse event reappear when the drug was re‐administered? | **+2** | **-1** | **0** |  |
| **5** | Are there alternative causes (other than the drug) that could on their own have caused the reaction? | **-1** | **+2** | **0** |  |
| **6** | Did the reaction reappear when a placebo was given? | **-1** | **+1** | **0** |  |
| **7** | Was the drug detected in blood (or other fluids) in concentrations known to be toxic? | **+1** | **0** | **0** |  |
| **8** | Was the reaction more severe when the dose was increased or less severe when the dose was decreased? | **+1** | **0** | **0** |  |
| **9** | Did the patient have a similar reaction to the same or similar drugs in any previous exposure? | **+1** | **0** | **0** |  |
| **10** | Was the adverse event confirmed by any objective evidence? | **+1** | **0** | **0** |  |
| **Total score (possible score -4 to 13)** | | | | |  |

**N.B:** if total score is a) ≥ 9 = definite ADR b) 5-8 = probable c) 1- 4 = possible d) ≤ 0 = doubtful ADR

**PLEASE! Fill the following information.**

1. Name of data collector…………………………………… Signature………….Date………..
2. Name of Supervisory …………………………………... Signature………… Date………..

**THANK YOU FOR YOUR GENIUNE INFORMATION!!!**
